# Prevalence of Preterm Premature Rupture of Membrane and Its Associated Factors among Pregnant Women Admitted in Debre Tabor General Hospital, North West Ethiopia: Institutional-Based Cross-Sectional Study

**DOI:** 10.1155/2020/4034680

**Published:** 2020-05-14

**Authors:** Dagne Addisu, Abenezer Melkie, Shimeles Biru

**Affiliations:** Department of Midwifery, College of Health Science, Debre Tabor University, Debre Tabor, Ethiopia

## Abstract

**Background:**

In Ethiopia, preterm premature rupture of membrane is defined as loss of amniotic fluid before the onset of labor in pregnancy >28 weeks of gestation but before 37 weeks. It is a significant cause of perinatal, neonatal, and maternal morbidity and mortality both in high- and low-income countries. Due to different factors associated with the quality of health care given and socioeconomic factors, the effect of preterm premature rupture of membrane is worsen in low-income countries. Little evidence is available about the problem in the study area. Therefore, this study was aimed to determine the prevalence of preterm premature rupture of membrane and its associated factors among pregnant women admitted in Debre Tabor General Hospital.

**Methods:**

Facility-based cross-sectional study was conducted. A total of 424 mothers were included in the study. Systematic random sampling was used to select study participants. A combination of chart review and interview was used to collect the data. Both descriptive and analytical statistics were computed.

**Result:**

The prevalence of preterm premature rupture of membrane was found to be 13.7%. Pregnant women with abnormal vaginal discharge (AOR = 5.30, 95% CI  = 2.07–13.52), urinary tract infection (AOR = 2.62, 95% CI = 1.32–5.19), history of premature rupture of membrane (AOR = 3.31, 95% CI = 1.32–8.27), vaginal bleeding (AOR = 2.58, 95% CI = 1.14–5.82), and mid-upper arm circumference <23 cm (AOR = 6.26, 95% CI = 3.21–12.20) were associated with preterm premature rupture of membrane.

**Conclusions:**

The prevalence of preterm premature rupture of membrane was high. Abnormal vaginal discharge, urinary tract infection, vaginal bleeding, previous premature rupture of membrane, and mid-upper arm circumference <23 cm were associated with preterm premature rupture of membrane. Thus, early screening and treatment of urinary tract infections and abnormal vaginal discharges were recommended to reduce the risk of preterm premature rupture of membrane.

## 1. Background

Preterm premature rupture of the membranes (PPROM) is defined as loss of amniotic fluid before the onset of labor in pregnancies before 37 weeks of gestation, which is characterized as a painless flow of fluid that escapes out of the vagina [1].

In Ethiopia, the lower limit of gestational age to define PPROM is different from other developing countries and defines PPROM as loss of amniotic fluid before the onset of labor during pregnancy after fetal viability (>28 weeks of gestation) but before 37 weeks of gestation. Rupture of membrane before 28 weeks of gestation is considered as previable or inevitable abortion [2].

The magnitude of PPROM varies in different countries and populations. It affects 3–4.5% of pregnancies globally [3, 4]. Evidences also discovered that PPROM accounts 3.1% in Brazil [5], 2.2 % in Manipur, India [4], 19.2% in China [3], 5.3% in Egypt [6], 3.3% in Nigeria [7], and 7.5 % in Uganda [8]. However, the magnitude is unknown in Ethiopia.

The exact cause of PPROM is unknown. However, different findings revealed that maternal ethnic origin, previous adverse pregnancy outcome, uterine over distention, smoking, low body mass index, genitourinary tract infection, maternal depression, pre-pregnancy stress, poor diet, assisted fertility, and periodontal diseases are the major contributing factors for PPROM [1, 5, 9, 10].

The burden of PPROM ranges from maternal and neonatal mortality and morbidity to countrywide economic loss due to drug expense, hospitalization, absenteeism from the work, and expense to the health professionals [1, 11].

PPROM is the primary cause of preterm deliveries and responsible for one-third of preterm births and 90% of neonatal death [3, 12]. It also increases the risk for neonatal resuscitation, intraventricular hemorrhage, infection, and respiratory distress syndrome [13, 14].

PPROM also leads to significant maternal complications such as puerperal infections, disseminated intravascular coagulopathy, placental abruption, operative delivery, chorioamnionitis, and psychological and lactation problems [15–17].

In Ethiopia, training manuals and guidelines were prepared to make health professionals experienced in managing and referring a woman with obstetric emergencies including PPROM [9]. Pregnant woman with PPROM is referred to hospital in which neonatal intensive care unit is available. The treatment of PPROM depends on the gestational age, presence of infection, and condition of the fetus. PPROM without chorioamnionitis is managed expectantly until 37 weeks of gestation. During expectant management, the patient will be admitted at hospital and followed closely. Vaginal examination will be avoided. The patient will have bed rest and IV hydration. Prophylactic antibiotics ampicillin 2 gm IV QID for 48 hours followed by 500 mg P.O. QID for 7–10 days plus erythromycin 500 mg IV QID for 48 hours followed by erythromycin 500 mg P.O. QID for 7–10 days will be given for the patient. If the gestational age is <34 weeks, betamethasone 12 mg IM every 24 hrs for 2 doses or dexamethasone 6 mg IV every 12 hrs for 4 doses will be given for lung maturity. In case of PPROM with chorioamnionitis, the pregnancy will be terminated regardless of gestational age [2, 18].

Although the prevalence and associated factors of PPROM were well studied in high-income countries, there is a scarcity of evidence in Ethiopia, particularly in the study area. Therefore, this study was aimed to determine the prevalence and associated factors of preterm premature rupture of membrane among pregnant women admitted in Debre Tabor General Hospital, North West Ethiopia. This finding is important to design evidence-based intervention and appropriate prevention strategies for reduction of PPROM in the study area.

## 2. Methods

### 2.1. Study Settings and Design

Facility-based cross-sectional study was conducted at the Department of Obstetrics and Gynecology of Debre Tabor General Hospital from March 14 to June 20, 2019. The hospital is located in the South Gondar zone, Amhara Regional State of Ethiopia. It is found at six hundred sixty-seven kilometers away from Addis Ababa, the capital city of Ethiopia.

Debre Tabor General Hospital gives service for more than five million people within its catchment area. The Department of Obstetrics and Gynecology has maternity, high-risk, and labor wards. The labor ward provides services for approximately three hundred eighty deliveries per month, and approximately eighty pregnant women were admitted in the high-risk ward per month.

### 2.2. Study Population

All pregnant women who were admitted in obstetric wards of Debre Tabor General Hospital between 28 and 36^+6^ weeks of gestation were the source population. This study included all pregnant women who were admitted in obstetric wards of Debre Tabor General Hospital during data collection period between 28 and 36^+6^ weeks of gestation. Pregnant women were admitted for various reasons such as (preeclampsia/eclampsia, APH, oligohydramnios, PPROM, preterm labor, GDM, DM, severe IUGR, superimposed preeclampsia, chronic hypertension, non-reassuring biophysical profile, severe anemia, IUFD, pyelonephritis, previous uterine rupture near term, toxic nodular goiter, and cardiac disease).

### 2.3. Measurement and Definition

#### 2.3.1. Preterm Premature Rupture of Membrane (PPROM)

In Ethiopia, PPROM is defined as loss of amniotic fluid before the onset of labor in pregnancies after fetal viability (>28 weeks of gestation) but before 37 weeks of gestation [2]. PPROM is classified into early PPROM (29–33^+6^ weeks) and late PPROM (34–36^+6^ weeks).

The diagnosis of PPROM was confirmed by clinicians through visualization of amniotic fluid passing from the cervical canal and pooling in the vagina or by using a basic pH test of vaginal fluid or ferning of dried vaginal fluid identified under microscopic evaluation.

#### 2.3.2. Anemia

A pregnant women whose hemoglobin level <11 g/dl was considered as anemic.

Gestational age was estimated using 1^st^ or 2^nd^ trimester (up to 24 weeks) ultrasound report and last normal menstrual period.

#### 2.3.3. Mid-Upper Arm Circumference (MUAC)

MUAC of each woman was measured at the midpoint between the tips of the shoulder and elbow of the left arm using nonelastic MUAC tapes. In this study, poor nutritional status of the mother was defined as MUAC <23 cm [1].

Maternal obstetric and medical problems such as abnormal vaginal discharge, GDM, UTI, and anemia were diagnosed by physicians during routine examination and were documented on the maternal medical chart.

### 2.4. Sample Size Determination

The sample size was determined using a single population proportion formula by assuming a 95% confidence level, marginal of error 5%, and prevalence of PPROM 50% since there are no previous similar studies. By adding 10% nonresponse rate, the final sample size was 424.

### 2.5. Sampling and Sampling Procedure

Systematic random sampling was applied to select study participants from labor, maternity, and high-risk wards, which was adopted from previous study [19]. The average number of pregnant women who were admitted in Debre Tabor General Hospital during the data collection period were estimated based on the previous admission, which was obtained by referring a six-month registration book/record prior to data collection. Around 840 pregnant women were admitted in labor, maternity, and high-risk wards in six months. The data were collected within a six-month duration. The sampling interval (*k*^th^ unit) was obtained by dividing the entire pregnant women (the total number of pregnant women who were admitted in six months) (840) by the desired sample size (total number of sample size) and it was approximately 2. The first woman was randomly chosen for the survey by the lottery method, and then every second woman who was admitted in the ward was recruited for the study.

### 2.6. Data Collection Tools and Procedures

Interview, chart review, and measurements were used to collect the data. Three BSc midwives and one supervisor were used for data collection and supervisory activity, respectively, after training was given for them. Structured interviewer-administered data collection formats were adapted and modified from different kinds of literature [1, 5, 6, 9, 10]. Questionnaires were categorized into sociodemographic characteristics, past and current obstetric and gynecological history, medical history, and behavioral factors. Data on respondent's specific questionnaires were collected by reviewing medical records and through interviewing the respondents. In addition, MUAC of each woman was measured at the midpoint between the tips of the shoulder and elbow of the left arm using nonelastic and nonstretchable MUAC tapes [1].

### 2.7. Data Management and Analysis

Data were entered into EPI data version 4.2 and then exported to SPSS version 23 for analysis. Descriptive statistics and binary logistic regressions were computed. Binary logistic regression was used to select variables associated with PPROM. In binary logistic regression, both bivariate and multivariable logistic regressions were computed. In bivariate analysis, independent variables with *p* value < 0.2 were selected as candidates for multiple logistic regressions. In multivariable logistic regression, statistical significant was considered at *p<*0.05. AOR and their 95% confidence interval (CI) were used to measure strength of the association. Backward stepwise logistic regression was applied. Finally, the data were presented with tables.

## 3. Results

### 3.1. Sociodemographic Characteristics of Respondents

A total of 424 pregnant women were enrolled in the study with a response rate of 100%. The mean age of the study participants was 29.76 years with standard deviation (SD) of ±6.239 years. Almost all (406 (95.8%)) of pregnant women were Amhara in ethnicity. Nearly three-fourths (311 (73.3%)) of pregnant women were from urban areas, and the majority (377 (88.9%)) of respondents were orthodox Christian follower (Table 1).

### 3.2. Reason for Admission

Nearly one-fourth (116 (27.36%)) of respondents were admitted for the indications of preeclampsia/eclampsia, and 58 (13.68%) of respondents were admitted for the indications of APH and PPROM (Table 2).

### 3.3. Past and Current Obstetrics-Related Characteristics of Respondents

The majority (381 (89.9%)) of respondents had ANC follow-up in current pregnancy, and nearly three-fourths (305 (71.9%)) of pregnant women were multigravida. Most of respondents (86.79%) had cephalic presentation, and 31 (7.1%) of pregnant women had history of PROM (Table 3).

### 3.4. Medical and Behavioral Characteristics of Respondents

Nearly one-fifth (86 (20.3%)) of pregnant women had UTI in current pregnancy, 28 (6.6%) had abnormal vaginal discharge, and 18 (4.2%) of pregnant women had gestational diabetes mellitus. All of pregnant women (100%) did not use cocaine, chat, and cigarettes (Table 4).

### 3.5. Prevalence of PPROM

The prevalence preterm premature rupture of membrane (PPROM) was found to be 13.67% (95% CI = 10.6–17.2).

### 3.6. Determinants of PPROM

The associations of PPROM with sociodemographic, obstetrical, medical, and behavioral characteristics of pregnant women were assessed. Abnormal vaginal discharge during pregnancy, residency, maternal age, vaginal bleeding in current pregnancy, average monthly income, MUAC of the mother, anemia, UTI, and previous history of PROM became significant at the level of 0.2 in the bivariate analysis. However, abnormal vaginal discharge, vaginal bleeding in current pregnancy, previous history of PROM, MUAC of the mother, and UTI remained significant and independently associated with PPROM in the multivariable analysis (Table 5).

## 4. Discussion

The prevalence preterm premature rupture of membrane (PPROM) was found to be 13.67% (95% CI = 10.6–17.2). This finding was higher than the study finding from Rio Grande in Brazil (3.1%) [5], tertiary care center in India (2.01%) [17], and Kampala International University teaching hospital in Uganda (7.5%) [8]. This discrepancy might be due to the difference between the quality of services they provided and socioeconomic status of study participants. In addition, two of the former studies were from developed countries in which good qualities of health care services were given for early screening and treatment of risk factors for PPROM. Furthermore, this study used data from a selected high-risk population which increases the magnitude of PPROM.

This finding was also higher than the study finding in Ain Shams Maternity Hospital in Egypt (5.3%) [6] and a tertiary hospital in Nigeria (3.3%) [7]. This discrepancy might be due to the difference in the accessibility and the quality of services in study settings. In addition, this study was conducted in the area where poor nutritional status was high. Furthermore, this study included selected high-risk populations which increase the magnitude of PPROM.

On the other hand, this finding was lower than the study findings in Jiangsu Province Hospital in China (19.2%) [3]. The difference could be attributed to the time gap between the studies. In addition, absence of behavioral risk factors for PPROM such as smoking, cocaine use, and alcohol consumption in the present study might attribute to this difference.

UTI during pregnancy was significantly associated with the development of PPROM. This finding was consistent with the study performed by Singh et al.[17]. The odds of PPROM was 2.62 times higher among those pregnant women with a history of UTI in pregnancy than those who did not have UTI (AOR = 2.62, 95% Cl = 1.32–5.19). Elevations of inflammatory mediators such as prostaglandins, cytokines, and proteinases in the local tissue play a causative role in disruption of fetal membrane integrity and in triggering uterine contractility. They are produced as a part of physiologic maternal defense mechanism in response to pathogens' invasion. The inflammatory mediators and production of matrix degrading enzymes and TNFs are involved in mechanisms of PPROM [20].

The present study also indicated that a significant association was noted between abnormal vaginal discharge and PPROM. The odds of PPROM was 5.30 times higher among pregnant women with abnormal vaginal discharge as compared to those who did not have abnormal vaginal discharge (AOR = 5.30, 95% C1 = 2.07–13.52). This finding was in agreement with the study findings in Mekelle city, Ethiopia [9]. Abnormal vaginal discharge is a common symptom of genital infections. Inflammatory cells produced by genital infections are involved in weakening of the fetal membranes among pregnant women thus causing PPROM [21].

In this study, previous history of PROM showed a significant association with PPROM. Pregnant women who had history of PROM were 3.31 times more likely to develop PPROM than those who did not have (AOR = 3.31, 95% Cl = 1.32–8.27). This finding was consistent with the study findings in the University of Calabar Teaching Hospital, Nigeria [22], and Mekelle city, Ethiopia [9]. This could be due to untreated genitourinary infection and a short cervical length (cervical incompetence). In addition, obstetric complications are highly recurrent by nature.

This study also indicated that pregnant women with MUAC <23 cm were significantly associated with PPROM. Pregnant women whose MUAC <23 cm were 6.26 times higher odds of developing PPROM than those ≥23 cm (AOR = 6.26, 95% Cl = 3.21–12.20). MUAC <23 cm during pregnancy indicates poor nutritional status. Nutritional deficiency particularly micronutrients deficiencies such as vitamin C or ascorbic acid affects collagen formation. Ascorbic acid protects the body against degenerative processes resulting from oxidative stress. In addition, it is important to strengthen and stabilize collagen by acting as an enzymatic cofactor. A dietary deficiency of micronutrients might lead to PPROM due to collagen weakness and capillary hemorrhage.

Women who had a history of vaginal bleeding in current pregnancy were 2.58 times more likely to develop PPROM as compare to their counterparts (AOR = 2.58, 95% CI = 1.14–5.82). This could be due to thrombin. Thrombin is formed when the tissue factor is released from decidual cells as a result of decidual hemorrhage, and it regulates genes involved in apoptosis and induces expression of inflammatory cytokines. This condition could result in tissue necrosis and degradation of the extracellular matrix.

We assessed hospital-admitted pregnant women (high-risk population) who might affect the actual prevalence of PPROM and its generalization to the whole target populations in the community as well as in the country as a whole. In addition, we used a small sample size which might affect the generalization. Another limitation is possible to selection bias. Furthermore, we did not collect data on some variables such as interpregnancy interval and body mass index.

## 5. Conclusions

Since this study was carried out in hospital-admitted pregnant women (high-risk population), we recommend large low-risk population-based studies in the study area as well as in the country as a whole to interpret differences between countries. Factors that tend to facilitate PPROM include abnormal vaginal discharge, UTI, vaginal bleeding during pregnancy, history of previous PROM, and MUAC of the mother <23 cm. Hence, improved nutritional statuses of pregnant women, early screening, diagnosis, and quick treatments of UTI and abnormal vaginal discharges were recommended to decrease PPROM.

## Figures and Tables

**Table 1:** Sociodemographic characteristics of pregnant women who were admitted in Debre Tabor general hospital, North West Ethiopia, 2019 (*n* = 424).

Characteristics	Frequency	Percent
Age (in years)		
15–19	60	14.2
20–34	247	58.3
≥35	117	27.6

Ethnicity		
Amhara	406	95.8
Others (Tigre and Oromo)	18	4.2

Residency		
Urban	311	73.3
Rural	113	26.7

Religion		
Orthodox	377	88.9
Muslim	39	9.2
Protestant	8	1.9

Educational status		
Nonformal education	105	24.8
Formal education	319	75.2

Occupational status		
Housewife	211	49.8
Civil servant	86	20.3
Merchant	89	21
Others (student, daily laborer)	38	9

Average monthly income (in birr)		
<1000 birr	163	38.4
1001–2000 birr	166	39.2
>2000 birr	95	22.4

MUAC of the woman		
<23 cm	67	15.8
≥23 cm	357	84.2

**Table 2:** Reasons (indications) of admission for respondents in Debre Tabor general hospital, North West Ethiopia, 2019 (*n* = 424).

Characteristics	Frequency	Percent
Indication for admission		
Preeclampsia/eclampsia	116	27.36
APH	58	13.67
Oligohydramnios	23	5.43
PPROM	58	13.67
Preterm labor	31	7.31
GDM	19	4.48
Diabetes mellitus	7	1.65
Sever IUGR	16	3.78
Superimposed preeclampsia	13	3.08
Chronic hypertension	11	2.59
Non-reassuring biophysical profile	21	4.95
Severe anemia	19	4.48
Intrauterine fetal death	12	2.83
Others	20	4.72

Others: pyelonephritis, previous uterine rupture near term, toxic nodular goiter, cardiac disease.

**Table 3:** Past and current obstetric characteristics of pregnant women who were admitted in Debre Tabor general hospital, North West Ethiopia, 2019 (*n* = 424).

Variables	Frequency	Percent
Gravidity		
Primigravida	97	22.9
Multigravida	305	71.9
Grand multigravida	22	5.2

Parity		
Nullpara	111	26.2
Primipara	129	30.4
Multipara	184	43.4

Gestational age		
29–33 weeks	129	30.4
34–36 weeks	295	69.6

ANC follow-up		
Yes	381	89.9
No	43	10.1

Vaginal bleeding in current pregnancy		
Yes	58	13.7
No	366	86.7

History of PROM		
Yes	31	7.3
No	393	92.7

History of preterm birth		
Yes	32	7.5
No	392	92.5

Type of pregnancy		
Singleton	394	92.9
Multiple	30	7.1

Polyhydramnios		
Yes	17	4
No	407	96

Presentation		
Cephalic	368	86.79
Breech	50	11.79
Shoulder	6	1.42

Labor pain		
Yes	31	7.3
No	393	92.7

**Table 4:** Medical and behavioral characteristics of pregnant women admitted in Debre Tabor general hospital, North West Ethiopia, 2019 (*n* = 424).

Variables	Frequency	Percent
Anemia		
Yes	27	6.4
No	397	93.6

UTI current pregnancy		
Yes	86	20.3
No	338	79.7

Abnormal vaginal discharge		
Yes	28	6.6
No	396	93.4

GDM		
Yes	19	4.5
No	405	95.5

Lifting heavy objects		
Yes	58	13.7
No	366	86.7

Falling in accident		
Yes	2	0.5
No	422	99.5

**Table 5:** Bivariate and multivariable association of PPROM and independent factors among pregnant women admitted in Debre Tabor general hospital, North West Ethiopia, 2019.

Variable	Preterm PROM		COR (95%)	AOR (95%)
	Yes	No		
Age				
15–19	14 (23.7%)	45 (76.3%)	1	1
20–34	31 (12.6%)	216 (87.4%)	0.46 (0.22–0.93)	0.44 (0.19–1.04)
≥35	13 (11%)	105 (89%)	0.39 (0.17–0.91)	0.43 (0.16–1.15)

UTI				
Yes	23 (26.7%)	63 (73.3%)	3.16 (1.74–5.71)	2.62 (1.32–5.19)^*∗*^
No	35 (10.4%)	203 (89.6%)	1	1

Vaginal bleeding				
Yes	12 (30.7%)	46 (79.3%)	1.81 (0.89–3.67)	2.58 (1.14–5.82)^*∗*^
No	46 (12.6%)	320 (87.4%)	1	1

Anemia				
Yes	7 (25.9%)	20 (74.1%)	2.37 (0.95–5.89)	2.05 (0.75–5.59)
No	51 (12.8%)	346 (87.2%)	1	1

Abnormal vaginal discharge				
Yes	13 (46.4%)	15 (53.6%)	6.76 (3.02–15.11)	5.30 (2.07–13.52)^*∗*^
No	45 (11.4%)	351 (88.6%)	1	1

MUAC of the mother				
<23 cm	28 (41.8%)	39 (58.2%)	7.82 (4.24–14.44)	6.26 (3.21–12.20)^*∗*^
≥23 cm	30 (8.4%)	327 (91.6%)	1	1

Monthly income				
<1000 birr	26 (16%)	136 (84%)	2.07 (0.90–4.80)	2.04 (0.78–5.31)
1001–2000 birr	24 (14.4%)	143 (85.6%)	1.82 (0.78–4.24)	1.37 (0.52–3.64)
>2000 birr	8 (8.4%)	87 (91.6%)	1	1

Previous PROM				
Yes	12 (38.7%)	19 (61.3%)	4.76 (2.17–10.45)	3.31 (1.32–8.27)^*∗*^
No	46 (11.7%)	347 (88.3%)	1	1

Residency				
Urban	38 (12.2%)	273 (87.8)	1	1
Rural	20 (17.7%)	93 (82.3%)	1.54 (0.85–2.78)	1.61 (0.79–3.29)

^*∗*^ *p* value < 0.05.

## Data Availability

The datasets used in this study are accessible from the corresponding author upon request.

## Abbreviations

ANC: Antenatal care
AOR: Adjusted odd ratio
APH: Antepartum hemorrhage
CI: Confidence interval
COR: Crude odd ratio
GDM: Gestational diabetes mellitus
MUAC: Mid-upper arm circumference
PPROM: Preterm premature rupture of membrane
PROM: Premature rupture of membrane
SD: Standard deviation
U/S: Ultrasound
UTI: Urinary tract infection.

## References

[1] Workineh Y., Birhanu S., Kerie S., Ayalew E., Yihune M. (2018). Determinants of premature rupture of membrane in Southern Ethiopia, 2017: case control study design. *BMC Research Notes*.

[2] Food, Medicine and Healthcare Administration and Control Authority of Ethiopia (2014). *Standared Treatment Guidelines for General hospitals*.

[3] Chandra I., Sun L. (2017). Third trimester preterm and term premature rupture of membranes: is there any difference in maternal characteristics and pregnancy outcomes?. *Journal of the Chinese Medical Association*.

[4] Mohan S. S., Thippeveeranna C., Singh N. N., Singh L. R. (2017). Analysis of risk factors, maternal and fetal outcome of spontaneous preterm premature rupture of membranes: a cross sectional study. *International Journal of Reproduction, Contraception, Obstetrics and Gynecology*.

[5] Hackenhaar A. A., Albernaz E. P., Fonseca T. M. V. D. (2014). Preterm premature rupture of the fetal membranes: association with sociodemographic factors and maternal genitourinary infections. *Jornal de Pediatria (Versão em Português)*.

[6] Abouseif H. A., Mansour A. F., Sabbour S. (2018). Prevalence and outcome of preterm premature rupture of membranes (PPROM) among pregnant women attending Ain Shams maternity hospital. *Egyptian Journal of Community Medicine*.

[7] Okeke T. C., Enwereji J. O., Okoro O. S., Adiri C. O., Ezugwu E. C., Agu P. U. (2014). The incidence and management outcome of preterm premature rupture of membranes (PPROM) in a tertiary hospital in Nigeria. *American Journal of Clinical Medicine Research*.

[8] Byonanuwe S., Nzabandora E., Nyongozi B. (2020). Predictors of premature rupture of membranes among pregnant women in rural Uganda: a cross-sectional study at a tertiary teaching hospital. *International Journal of Reproductive Medicine*.

[9] Assefa N. E., Berhe H., Girma F. (2018). Risk factors of premature rupture of membranes in public hospitals at Mekele city, Tigray, a case control study. *BMC Pregnancy and Childbirth*.

[10] Zhou Q., Zhang W., Xua H. (2014). Risk factors for preterm premature rupture of membranes in Chinese women from urban cities. *International Journal of Gynecology and Obstetrics*.

[11] Landon M. B., Galan H. L., Jauniaux E. R. (2020). *Obstetrics: Normal and Problem Pregnancies E-Book*.

[12] Oza S., Lawn J. E., Hogan D. R., Mathers C., Cousens S. N. (2014). Neonatal cause-of-death estimates for the early and late neonatal periods for 194 countries: 2000–2013. *Bulletin of the World Health Organization*.

[13] Rajan R., Menon V. (2016). Preterm premature rupture of membranes: correlates and pregnancy outcome in a tertiary care setting. *International Journal of Research in Medical Sciences*.

[14] Endale T., Fentahun N., Gemada D., Hussen M. A. (2016). Maternal and fetal outcomes in term premature rupture of membrane. *World Journal of Emergency Medicine*.

[15] Vishwakarma K., Patel S. K., Yadav K., Pandey A. (2015). Impact of premature rupture of membranes on maternal & neonatal health in Central India. *Journal of Evidence Based Medicine and Healthcare*.

[16] Idrisa A., Pius S., Bukar M. (2019). Maternal and neonatal outcomes in premature rupture of membranes at University of Maiduguri Teaching Hospital, Maiduguri, North-Eastern Nigeria. *Tropical Journal of Obstetrics and Gynaecology*.

[17] Singh T. D., Usham R., Kamei H. (2015). Preterm prelabour rupture of membrane 1 year study. *Journal of Evolution of Medical and Dental Sciences*.

[18] Minsitry of Health (2010). *Managment Protocol for Selected Obstetric Topics Addis Abeba Federal Democratic Repuplic of Ethiopia*.

[19] Addisu D., Asres A., Gedefaw G., Asmer S. (2018). Prevalence of meconium stained amniotic fluid and its associated factors among women who gave birth at term in felege hiwot comprehensive specialized referral hospital, North West Ethiopia: a facility based cross-sectional study. *BMC Pregnancy and Childbirth*.

[20] Tchirikov M., Schlabritz-Loutsevitch N., Maher J. (2018). Mid-trimester preterm premature rupture of membranes (PPROM): etiology, diagnosis, classification, international recommendations of treatment options and outcome. *Journal of Perinatal Medicine*.

[21] Nakubulwa S., Kaye D. K., Bwanga F., Tumwesigye N. M., Mirembe F. M. (2015). Genital infections and risk of premature rupture of membranes in Mulago Hospital, Uganda: a case control study. *BMC Research Note*.

[22] Emechebe C., Njoku C. O., Anachuna K., Udofia U. (2015). Determinants and complications of pre-labour rupture of membranes (PROM) at the University of Calabar Teaching Hospital (UCTH), Calabar, Nigeria. *Scholars Journal of Applied Medical Sciences*.

